# Short-distance detectability in camera trap surveys: implications for population assessment

**DOI:** 10.1007/s10661-026-15272-7

**Published:** 2026-04-17

**Authors:** Stefano Focardi, Isaia Maglia, Pietro Pontiggia, Barbara Franzetti

**Affiliations:** 1 Istituto dei Sistemi Complessi del CNR, Via Madonna del Piano, 10, Sesto Fiorentino (FI), 50019 Italy; 2 Italian Institute for Environmental Protection and Research, Rome, Italy; 3 Department of Biology, University of Rome Tor Vergata, Rome, Italy

**Keywords:** Camera trap, Detectability, Population assessment, Wild boar, *Sus scrofa*

## Abstract

Monitoring wildlife populations is essential for conservation and management. Many statistical methods are now available for estimating population density based on camera trapping when animals are unmarked and not individually recognisable. Several of these methods assume that the detection probability is known, or rely on detection functions derived from distance sampling theory, which assume that animals are perfectly detectable when in the immediate vicinity of the camera trap. We test this last assumption on the wild boar (*Sus scrofa*) in the Mediterranean forest of Castelporziano (Roma, Italy), carrying out three experiments. (1) We evaluate how detection probability varies with distance (0–6 m) from camera traps using one experimental and three control cameras across 12 sites. (2) We assess the impact of imperfect detectability near camera traps on population estimates by conducting a survey with 63 camera traps deployed in a systematic-random design, in the same habitats and season as in experiment 1; we compared survey results when we substituted the detection function derived from experiment 1 with the standard detection functions used in the Random Encounter Model. (3) We compare the Bolyguard SG2060-K and Uovision UV595-HD (used in experiments 1 and 2) with the high-performance Browning Spec Ops HP5 to assess if the camera trap model may have affected our findings. We found that (1) the detection probability is less than 1 close to the camera trap; (2) the Random Encounter Model underestimated population density by 17%; and (3) there are no significant differences in performance among the three camera trap models when monitoring wild boars. These findings indicate that camera trap-based population models may yield biased estimates unless they account for detection probabilities below 1.

**Supplementary Information:**

The online version contains supplementary material available at 10.1007/s10661-026-15272-7.

## Introduction

The last 10 years have seen a substantial increase in the use of remote cameras in ecological research (Delisle et al., 2021; Rovero et al., 2013). Camera trapping is widely used for estimating the probability of species occurrence (MacKenzie et al., 2006) and can be used for describing variations in animal behavior as a function of environmental conditions and human impact (e.g., Caravaggi et al., 2020).

Estimating the size of wildlife populations generates critical data for conservation and management. When individual recognition is possible through natural markings (as in many felids and other species of conservation concern) or tags, camera traps can provide an appropriate method to estimate population abundance (e.g., jaguar, Borchers et al., 2014; Amur tiger, Xiao et al., 2016; snow leopard, Oberosler et al., 2022). However, since individuals of many species are not readily recognizable, there has been an extensive and rapid development of camera trap methods designed specifically for estimating abundance of “unmarked” populations. These include: the Random Encounter Model (REM, Rowcliffe et al., 2008), the Time To Event model (TTE, Moeller et al., 2018), the Random Encounter and Staying Time model (REST, Nakashima et al., 2018), the Time In Front of the Camera (TIFC) (Becker et al., 2022), the Camera Trap Distance Sampling (CT-DS) (Howe et al., 2017), and N-mixture models (Royle, 2004).

Detectability, the ability to detect an animal when present, affects precision, accuracy, and efficiency of camera trap surveys. Variation in detectability occurs across species, spatial contexts, and temporal scales (O’Connor et al., 2017).

Each camera trap (CT) operates within a defined detection zone (Nakashima et al., 2018; Rowcliffe et al., 2011), representing the area within its field of view where detectability is maximized. While the field of view is an angular parameter determined by the camera’s lens characteristics, the detection zone incorporates distance-based boundaries through left- and right-truncation (minimum and maximum detection distances, respectively). Nakashima et al., (2018) and Carswell et al., (2025) provide useful descriptions of how to calculate the detection zone. To correctly delineate the detection zone, several CT methods, including REM, TIFC, and CT-DS, rely on the theoretical framework of point transect sampling (Buckland et al., 2015). The detection probability (*P*ₐ) and radius (*r*) (also referred to as the effective detection distance) are fundamentally related through the following equation (Hofmeester et al., 2019):

1 $$r=w \cdot \sqrt(P_{a})$$

where *w* is the truncation distance and *P*_a_ is the expected detection probability for an animal within distance *w* of the camera (Buckland et al., 2015). When the detection probability is perfect (*P*ₐ = 1), the entire area between the camera trap and distance *w* constitutes the effective detection zone. However, when detection is imperfect (i.e., *P*ₐ < 1), the effective detection radius must be adjusted such that the expected number of undetected animals within distance *r* is equal to the expected number of detected animals between *r* and *w*.

Findlay et al., (2020) and DeWitt & Cocksedge (2023) proposed a clear framework for understanding the detection process in CT studies, which hinges on a series of conditional probabilities: (1) the animal must encounter the CT, (2) the trigger mechanism must be activated, (3) the CT must successfully capture an image of the animal, and (4) image quality must be sufficient to identify the animal. The likelihood of an encounter depends on survey design, such as the use of bait or strategic CT placement. The ability of the CT to trigger relies on its technological features, particularly on the array of passive infrared (PIR) sensors, which detect temperature differences caused by an animal moving across the sensor array. The effectiveness of PIR sensors increases when an animal is closer to the camera, as its apparent size is larger, making the heat signature more detectable. Consequently, CTs are generally more effective at detecting larger animals, due to their greater surface area relative to the sensor array. Research, including studies by Heiniger & Gillespie (2018); Howe et al., (2017); Jacobs & Ausband (2018); Mason et al., (2022), and Rowcliffe et al., (2011), shows that detection probability rises with larger body size and decreases with greater distance from the CT, as the animal’s heat signature diminishes. Additionally, the relationship between the PIR sensor array and the animal can be influenced by factors such as deployment height and tilt, environmental temperature, and by the target species, as noted by Apps & McNutt (2018); Kelly & Holub (2008), and Welbourne et al., (2016). Together these variables shape the ability of the CT to detect wildlife effectively.

Trigger probability, a key component of detection, is also affected by distance, though through a different mechanism. It is influenced by both the apparent size of the animal—larger sizes typically resulting from closer proximity to the sensor array—and the angular speed of the animal, with slower movements enhancing detection. Thus faster-moving animals can outpace the trigger mechanism, particularly in the immediate proximity of the CT. Technical features and settings of CTs significantly affect performance, and species-specific movement patterns also play a role. There are two possible outcomes from the combination of these factors: (1) detectability decreases monotonically with distance from the CT or (2) CT may exhibit reduced detection efficiency for animals passing in very close proximity to the sensor. Consequently, detection probability typically reaches a maximum at an intermediate distance from the CT and then declines monotonically with increasing distance.

Monotonically decreasing functions, such as the half-normal and hazard-rate models, are commonly used in detection analyses. This distance-dependent detection pattern has been consistently observed across diverse environments and species of varying sizes and movement types (Rowcliffe et al., 2011; Howe et al., 2017; Pal et al., 2021; Cappelle et al., 2021; Corlatti et al., 2020, among others).

Direct estimates of detectability near CTs are scarce but a close look at the literature reveals multiple cases where detection probability near CTs is less than 1. Becker et al., (2022) used a double-platform method to show that reindeer detectability within 5 m of the CT was nearly certain (95%). Based on these findings, they suggested that animals of similar size likely exhibit comparable detection rates. DeWitt & Cocksedge (2023) further explored detectability as a function of distance by using human observers as proxies for differently sized animals. Their analysis revealed that the most parsimonious detection function was a half-normal logistic mixture, indicating that detectability declines close to the CT. Specifically, detectability began to decrease beyond approximately 6 m (with some variation across experimental conditions). While some scenarios showed consistently high detection near the CT, others exhibited a gradual decline starting at 4–6 m and approaching zero at greater distances. Rowcliffe et al., (2011) reported a reduced detectability near CTs for small- and medium-sized mammals in Barro Colorado Island (Panama). Moreover, in several CT-DS surveys, researchers were forced to apply left-truncation because of a lack of detections near the CT (e.g., Cappelle et al., 2021; Mason et al., 2022).

As previously stated, many estimation methods using CT data are based on point sampling theory which assumes objects at the sampling point are detected with certainty (*h*(0) = 1) (Buckland et al., 2015). This is directly analogous to the *g*(0) = 1 assumption in line transect sampling, where detection on the line is assumed to be certain. However, there are documented cases where this assumption may fail (Borchers & Cox, 2017). As stressed by Wearn et al., (2022), even when accounting for imperfect detection via Distance Sampling, one must ensure that detection is guaranteed at the sampling point (i.e., immediately in front of the CT)**.** Failure to meet this requirement results in downward-biased population estimates. To address this issue, Rowcliffe et al., (2011) developed a logistic mixture model to account for reduced detectability near camera traps. However, this approach remains limited, as estimates are inherently unreliable if detectability is unknown at any point. This gap is addressed by methods like Mark-Recapture Distance Sampling, which allow for a formal correction when *h*(0) < 1 (Buckland et al., 2015).

The wild boar (*Sus scrofa*) presents unique challenges for wildlife management across its global range. As a highly adaptable species native to Europe, Asia, and North Africa, with introduced populations in the Americas and Australia, its “native invader” status (Carey et al., 2012) creates significant management issues. Furthermore, the numerical increase of wild boar exerts significant ecological and economic pressures which range from ecosystem engineering that threatens biodiversity to agricultural damage and synanthropy. This population growth also compromises human activities by increasing the risk of traffic accidents, the spread of zoonotic diseases, and the persistence of African Swine Fever (ASF) panzootics.

Unlike typical ungulates, wild boars exhibit three distinctive characteristics that compound these challenges: an explosive population dynamics driven by unusual life history traits (high reproductive rates and short lifespans (Focardi et al., 2008) and extreme interannual population fluctuations tied to food availability (e.g., masting events; Gamelon et al., 2021). 

These biological and ecological traits render traditional habitat suitability models ineffective, creating an urgent need for direct population assessment. However, this approach introduces its own challenges: the species’ secretive behavior makes reliable surveying difficult (Focardi et al., 2020). As a result, researchers and managers are increasingly relying on camera trapping and associated analytical frameworks, such as REM (ENETWILD-consortium et al., 2023), to generate population estimates. Yet detection biases inherent to CT techniques can lead to substantially inaccurate population estimates, potentially undermining the effectiveness of management and control efforts. Given the high reproductive rates and considerable socio-economic impacts of wild boar, underestimating population size can result in serious management failures, including insufficient mitigation of crop damage or misjudging infection risks in the context of transmissible diseases.

Our aim is to test a fundamental assumption common to several CT population estimation methods: that detectability near the CT is certain (*h*(0) = 1). We adopted a three-stage approach. First, we conducted controlled experiments to assess variations of detectability at close range to CTs (range 0–6 m). Second, we compared these experimental results with field data from a CT survey—adopting the Random Encounter Model (Rowcliffe et al., 2008)—conducted in the same habitats and season as the first experiment to quantify potential biases introduced by imperfect detection near the CT. Finally, to enhance the generalizability of our findings, we compared the performance of the two CT models used in the two experiments against a reference high-end model known for superior detection capabilities.

## Materials and methods

**Fig. 1:**
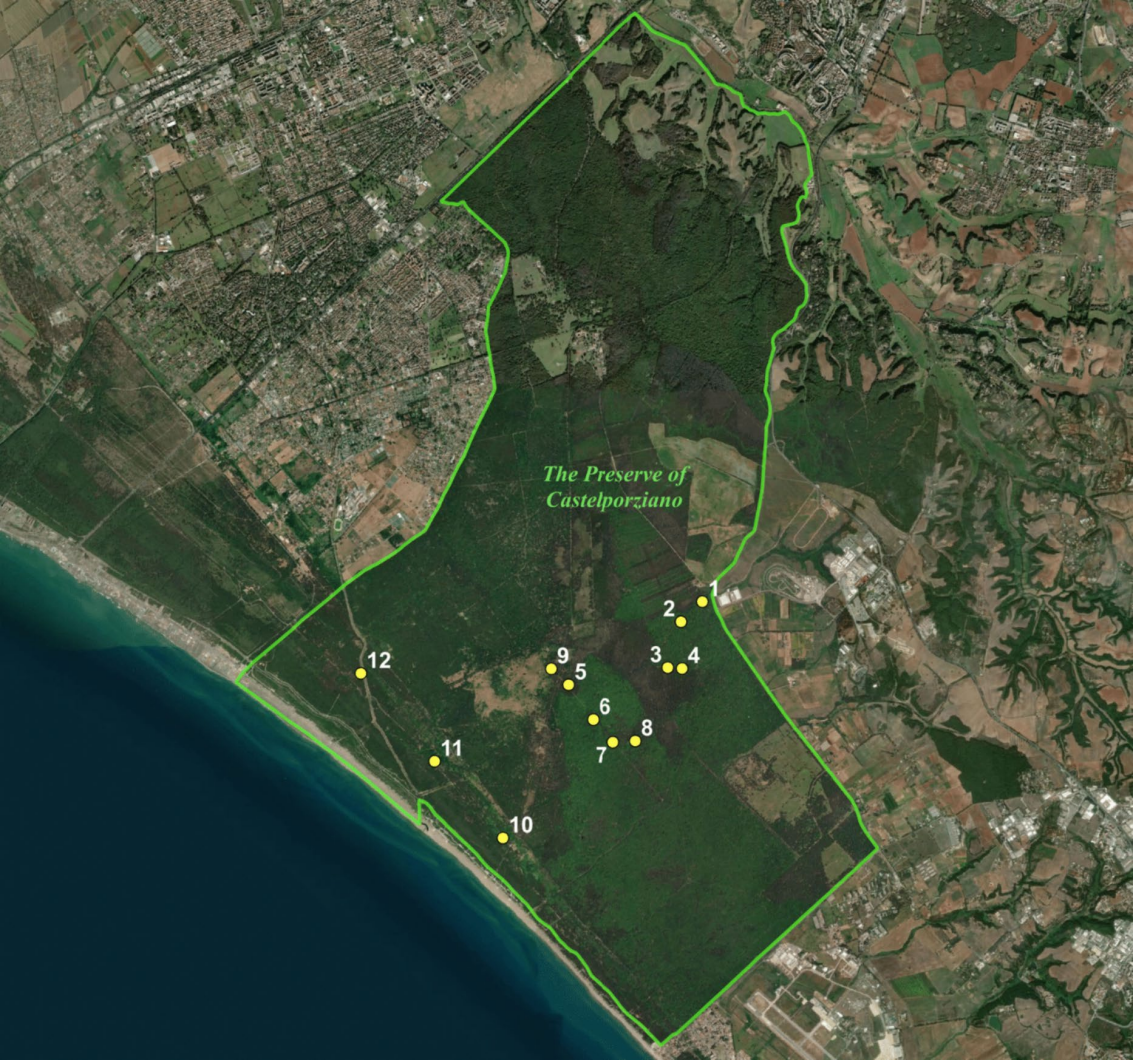
The Preserve of Castelporziano (Rome, Italy). Yellow dots represent the location of the experimental plot in the CT Detection experiment (2.1)

The study was conducted in the fenced 60 km^2^ Preserve of Castelporziano (Roma, Italy, Fig. 1). This area mainly comprises natural oak woods, including both evergreen (*Quercus ilex* and *Q. suber*) and deciduous (*Q. cerris* and *Q. frainetto*) species as well as Mediterranean maquis. Mixed or pure forests of domestic pine (*Pinus pinea*) and pastures are also present (for a full description of the area see Giordano, 1992). Our study focused on the four main habitats of the Preserve (*Echinopo–Quercetum*, *Viburno–Quercetum ilicis*, Mediterranean maquis and pine plantations, Giordano, 1992; Pignatti et al., 2001), excluding pastures, cultivated and fenced areas. Climate is typically Mediterranean with dry summers and rainfall concentrated in October–November. Mean annual rainfall is 805 ± 256 mm (Aromolo et al. 2015) and mean monthly temperatures range from 8.74 ± 1.38 °C in January to 24.95 ± 1.03 °C in August. Winters are mild and snowless.

We conducted: (1) an experimental trial (denoted by subscript EXP) to assess the detection probability of camera traps for wild boar in the range of 0–6 m; (2) a survey (denoted by subscript SUR) to test the application of the Random Encounter Model as implemented in the Agouti platform (Casaer et al., 2019), which is commonly used in studies of this type (e.g., ENETWILD-consortium et al., 2023); and (3) an experimental comparison of the detection performance of the CTs used in studies (1) and (2) against a benchmark camera trap of better technological quality.

### Detection experiment

We conducted the detection experiment at 12 plots in the study area (Fig. 1). We deployed 4 CTs per plot, for a total of 48 units. The study was divided into two sampling periods: an initial pilot trial (7–22 May 2022) followed by a main trial (12 November–11 December 2022).

We used two different CT models:

1. Bolyguard SG2060-K. Trigger speed: < 1 s; declared FOV: 57°; measured FOV: 46° (Product details: https://www.bolymedia.com/index/Cameras_detail/id/23).
2. Uovision UV595-HD. Trigger speed: 0.65 s; declared FOV: 52°; measured FOV: 37,3° (Product details: http://www.uovision.com/Data/uovision/upload/file/20171102/UV595%20HD%20user%20manual-1.pdf).

We empirically determined the actual field of view (FOV) by placing reference targets at known distances from each CT.

**Fig. 2:**
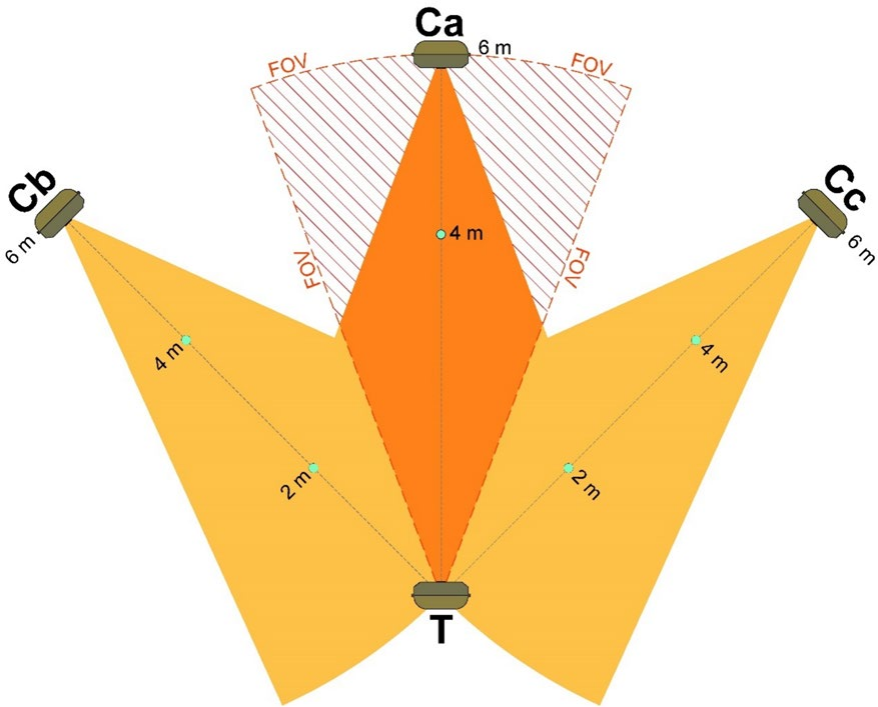
The trial layout made use of 4 camera traps and 5 plastic sticks (indicated by small green dots) to measure distances relative to the camera test (T). FOV indicates the limits of the field of view of the test CT. The control CTs are labelled Ca, Cb, and Cc. The hatched areas near Ca are likely visible only to T, which may inflate the estimated detection probability around 6 m and might explain instances where only the test CT detected animals. Animals observed by Cb or Cc outside the FOV of T were excluded from the analysis. The central orange area is where there is at least one CT able to detect animals

**Fig. 3:**
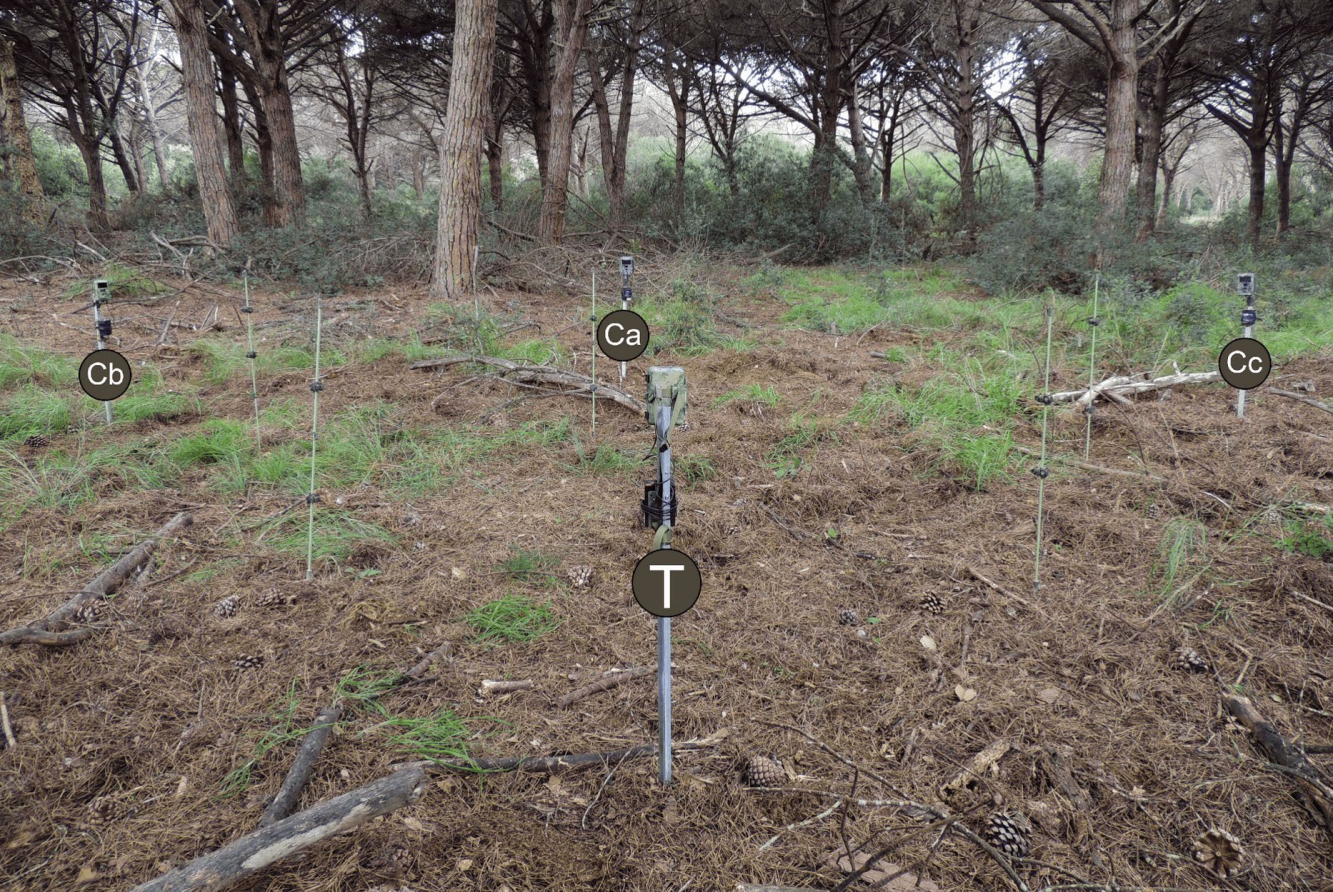
A picture of the trial layout. The overlapped label T indicates the test CT and the labels Ca, Cb, and Cc the three control CTs

We employed a balanced design with Bolyguard CTs surveying half of the test locations and Uovision CTs monitoring the remaining half. All CTs were mounted 90 cm above ground with a 10° downward tilt to optimize detection of approaching animals. We placed markers at 2-m intervals along the detection zone boundaries and centreline (Fig. 2). These plastic reference stakes enabled us to track animals moving through the CT detection zone (Fig. 3). Our methodology follows Hofmeester et al., (2019) and Palencia et al., (2021).

**Fig. 4:**
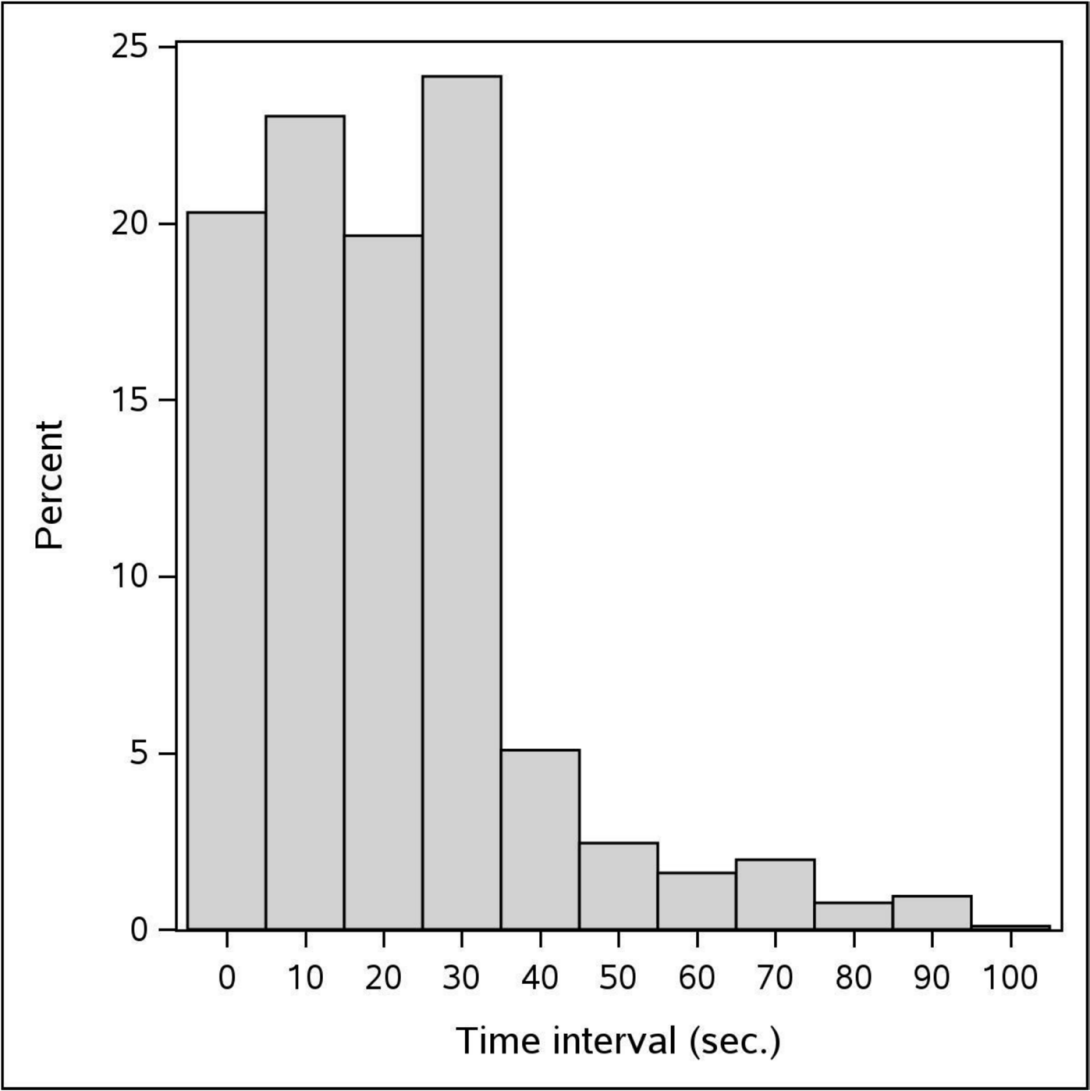
Histograms of *θ* distribution for the wild boar in the Preserve of Castelporziano (Rome, Italy). A threshold has been defined by selecting the midpoint of the first bin after the abrupt decrease in the number of cases (40 s)

For each detection of wild boars, we recorded: (1) distance from the test CT, (2) time of detection, (3) group composition, and (4) group size. To distinguish independent events from sequential detections of the same animal(s), we implemented a temporal threshold (*θ*) approach. Detections occurring within *θ* seconds of each other across different CTs were considered part of the same event (hereafter termed a “sequence”). Although ideal detection would show perfect synchronization across all 4 CTs, when an animal enters the detection zone, practical limitations, such as the direction of movement and speed, create time lags between detections by the different CTs. Additionally, animals moving continuously through overlapping fields of view can generate extended detection series. These two factors could artificially inflate detectability estimates for the test CT. To address this issue, we calculated time intervals between sequential detections and generated an interval histogram (Fig. 4). This allowed us to determine that a threshold of *θ* = 40 s provided an appropriate interval to define independent observation events. Below 40 s, we observed a high frequency of related detections, which decreased to a minimum at 40 s, thus allowing us to differentiate between correlated and independent detections. Unlike standard applications that aim to distinguish individuals, our primary concern was ensuring that CTs reset properly between independent capture events into the FOV. We validated this approach through visual inspection of a representative sample of sequences. The number of sequences represents the sample size for our analyses.

For each independent event in which an animal crossed the FOV of at least one CT, the detection probability (*p*_*exp*_) of the test CT was calculated as the proportion of animals it successfully detected out of the total number of animals known to have been present (i.e., detected by either the test or the control CTs). The overall group size (*group*) was determined by summing the maximum number of animals in each age and sex class (piglets, male and female subadults and adults) recorded across the four CTs.

Statistical analyses were conducted in SAS 9.4 (SAS Institute, 2010). Detection probability *p*_*exp*_ was modelled by logistic regression to ensure that it ranged from 0 to 1. All analyses were conducted using PROC GLIMMIX, employing the Laplace method with a binomial distribution and a logit link.

The following covariates were recorded:

1. The minimum distance between the test CT and a group of animals, referred to as *distance*, calculated relative to the test CT for both test and control CTs. To ensure consistency, we measured the distance when the group first entered the detection zone, as animals may approach the camera at varying distances before being detected (Rowcliffe et al., 2011). For individual animals, distance was simply the distance from the test CT to the animal. For a group of animals, it was the distance from the test CT to the closest animal in the group;
2. The time of the day, *dn*, indicated whether the observation occurred during the day (*dn* = D, 06:00–18:00) or at night (*dn* = N, 18:00–06:00);
3. The habitat type, denoted *habitat* (1 = *Echinopo–Quercetum*, 2 = *Viburno–Quercetum ilicis*; 3 = Mediterranean maquis; 4 = pine plantations);
4. The local canopy obstruction, *CanOb*, allowed us to test the hypothesis that vegetation cover may reduce detection rates. To provide an objective evaluation of vegetation obstruction, we modified the approach developed by Rutten et al., (2015) and used by Focardi et al., (2020) for a speedy evaluation of obstruction in each survey area. Since we were interested in wild boar, we positioned a rangefinder 55 cm high close to the test CT. In denser habitats, the rangefinder recorded shorter distances to the nearest obstacle. Measurements were taken at 45° intervals, and for each experimental site, we calculated the average *CanOb* value;
5. The group size, *group*;
6. The camera model, *CTmodel* (Uovision or Bolyguard);

Candidate models were generated as follows. We began with a full additive model containing all covariates (without interactions):

$$p_{EXP}=\mathrm{intercept} + r_{1}distance + r_{2}dn + r_{3}habitat + r_{4}CanOb + r_{5}group + r_{6}CTmodel + e,$$

where* r* represents the regression coefficient for each covariate and *e* is the error term. Categorical variables included *dn, habitat* and *CTmodel*. We sequentially simplified this model by removing covariates in the following order: *CTmodel, group, CanOb, habitat,* and *dn*. Based on existing literature, we assumed *distance* to be a critical factor influencing detectability. To assess its importance, we re-ran the models above, excluding *distance*. An intercept-only model was also evaluated. The 12 experimental plots were included as a random effect. Model selection was based on AICc (Burnham & Anderson, 2002).

To visualize the relationship between *p*_*EXP*_ and *distance*, we plotted the predicted detection function using the average values for *CanOb* (9.88) and *group* (1.6), while setting *habitat* = 1 (the most common habitat type) and *dn* = N (as most observations occurred at night). Predicted observations were plotted to reflect the sample size. For better readability in cases of overlapping observations, we applied the JITTER function in PROC SGPLOT. Mean detectability and its standard error were calculated using predicted values. To illustrate the distribution of observed data, we created a 100% stacked bar chart with three categories: no detection (*p*_*EXP*_ = 0), perfect detection (*p*_*EXP*_ = 1) and partial detection (0 < *p*_*EXP*_ < 1, denoted as *p*_*EXP*_ = 0.5, for brevity).

### Study area survey

**Fig. 5:**
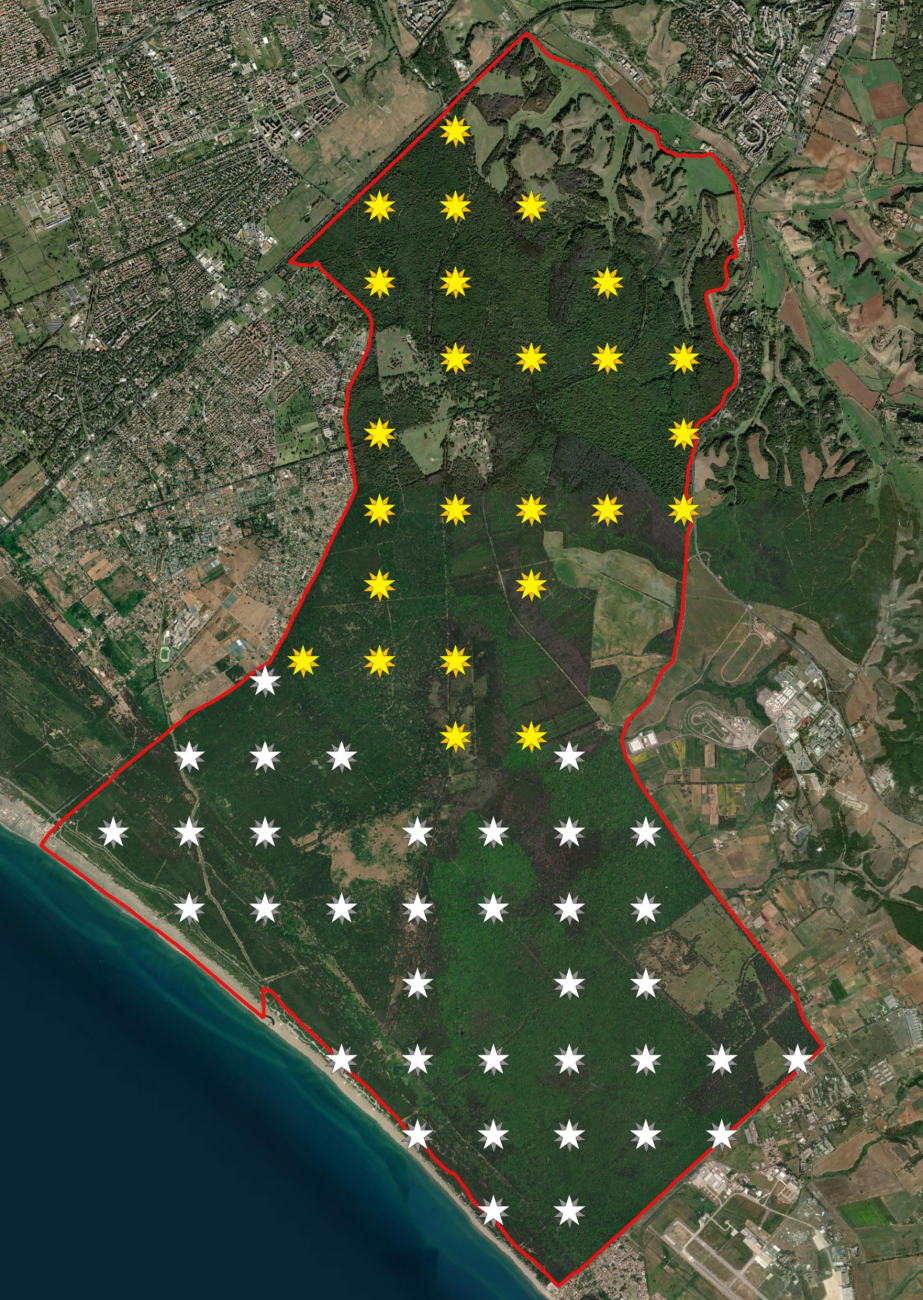
Distribution of camera traps during the REM survey in the Preserve of Castelporziano (Rome, Italy). Colors characterise the two survey areas (yellow: north sector, white: south sector)

To evaluate how detection probability influences REM estimates, we conducted a CT survey following ENETWILD protocols as implemented via the Agouti platform (https://agouti.eu/). We deployed 63 CTs across the four selected habitats, using a randomized systematic sampling design (Fig. 5). Sampling began in the northern area (Fig. 5, yellow symbols) from 15 September to 1 October 2022, after which the CTs were relocated to the southern area (Fig. 5, white symbols) for an additional 15 days of sampling (2–17 October 2022).

At each placement, we collected a series of calibration images following Agouti methodology, which enabled calculation of animal-to-camera distances. Using these data, we estimated REM parameters, including the daily range, effective detection radius (*r*_*SUR*_), and the angle of the effective detection zone.

We selected the most parsimonious detection function, either hazard-rate or half-normal, both strictly monotonically decreasing, with or without adjustment terms (cosine order 2, hermite order 4, or polynomial order 4). To align with the results of experiment 1, data were truncated at 6 m. From *p*_*EXP*_, we derived the experimental detection radius (*r*_*EXP*_, Eq. 1) and compared it with the effective detection radius derived from the survey (*r*_*SUR*_). Using the additional REM parameters—trapping rate, daily range, and detection angle—we calculated density estimates for both the experimental trial and the survey and evaluated the level of agreement between the two approaches.

### CT comparison

We compared the performance of two camera models, the Uovision and Bolyguard (trigger speed of 0.65 and 1 s, respectively), used in experiments 2.1 and 2.2, against a newer, more advanced camera model, the Browning Spec Ops HP5 (Product details: https://browningtrailcameras.com/products/spec-opselite-hp5), which was classified as high-grade by McHenry et al., (2025). The Browning CT features a 0.1 s trigger speed, which is faster than the other two models, and a larger declared and measured FOV (53.7°).

Sampling lasted 31 days and was conducted at two locations, each equipped with nine CTs (Table 1). The CTs were mounted on three stakes positioned 1 m apart, at three different heights (upper 60 cm, medium 35 cm, and low 10 cm) (cf. McIntyre et al., 2020 for a similar approach). Each camera was placed at varying heights to account for potential positional effects. To control for the influence of stake position (left, centre, or right) on detection probability, the stakes were rotated every 10 days. This balanced sampling design ensured that all three CT models were exposed to identical conditions regarding stake position and height by the end of the trial.

**Table 1:** The sampling program was conducted in two different locations in order to compare three CT models. The heights of CT placement were 60 cm (H), 35 cm (M), and 10 cm (L)

Operating period		Position	Stake		
Start	End		Left	Medium	Right
31/10/2024	10/11/2024	H	Browning	Boly	Uovision
		M	Uovision	Browning	Boly
		L	Boly	Uovision	Browning
10/11/2024	20/11/2024	H	Uovision	Browning	Boly
		M	Boly	Uovision	Browning
		L	Browning	Boly	Uovision
20/11/2024	30/11/2024	H	Boly	Uovision	Browning
		M	Browning	Boly	Uovision
		L	Uovision	Browning	Boly

Sequences, as defined in the “Detection experiment” section, were used as the sampling units. To compare the performance of the three CT models, we evaluated three dependent variables: (1) group size (the maximum number of animals observed during a sequence), (2) sequence duration (the time from the start to the end of a sequence), and (3) the number of independent wild boar detections.

To investigate differences in detected group size between CT models, we applied a Poisson regression with a log-link function. For sequence duration, which is strictly positive, we used a gamma regression with a log-link function. Both analyses were performed using PROC GENMOD. Finally, for the number of detections, we conducted a *χ*^2^ test using PROC FREQ.

## Results

In the CT detection experiment (“Detection experiment” section), we recorded 753 sequences, with model selection results presented in Table 2. The top four models consistently include the variable *distance*, emphasizing its influence on detectability. Models excluding distance exhibit much higher AICc values than corresponding models that include it; for instance, model1 and model7 show a ΔAICc = 117.85, which underscores the fundamental importance of distance. The covariates *dn* and *habitat* are included in the top models, whereas *CanOb*, *group*, and *CTmodel* are not.

**Table 2:** Model selection for the influence of different covariates (*distance*, *dn, habitat, CanOb, group,* and *CTmodel)* on detection probability (*p*_*EXP*_)*,* in CT experiment 2.1. The table displays the terms included in the model and the AICc. The number of parameters in each model is given by the number of covariates plus 1. The retained model is in bold

Model	AICc	Covariates					
1	1113.38	*distance*	*dn*	*habitat*	*CanOb*	*group*	
2	1115.26	*distance*	*dn*	*habitat*	*CanOb*	*group*	*CTmodel*
3	1115.44	*distance*	*dn*	*habitat*	*CanOb*		
4	1128.33	*distance*	*dn*	*habitat*			
5	1222.08	*distance*	*dn*				
6	1229.57		*dn*	*habitat*	*CanOb*		
7	1231.23		*dn*	*habitat*	*CanOb*	*group*	
8	1232.69		*dn*	*habitat*	*CanOb*	*group*	*CTmodel*
9	1241.54		*dn*	*habitat*			
10	1243.93	*distance*					
11	1389.71		*dn*				
12	1427.80						

The estimated coefficients for the best model (Model 1, Table 3) confirm that distance significantly impacts detectability, and detection probabilities were lower at night compared to daytime. Additionally, notable differences in detection probability were observed across habitats. Both *CanOB* and *group* are statistically significant. While the negative coefficient for *CanOB* was unexpected, it is consistent with the negative coefficient observed in habitat 4 (pine plantations).

**Table 3:** Estimated coefficients of the variables of the retained models (see the model selection in Table 2). Habitat = 3 and dn = N are used as the reference level for the two categorical variables. DF = 709

Effect	Levels	Estimate	Standard error	*t*	Pr >|t|
Intercept		0.74	0.63	1.18	0.24
*distance*		0.68	0.068	10.05	<.0001
*dn*	D	0.45	0.22	1.99	0.047
	N	0			
*habitat*	1	0.14	0.34	0.40	0.69
	4	− 1.43	0.36	− 4.02	<.0001
	2	2.80	1.04	2.70	0.007
	3	0			
*CanOb*		− 0.23	0.05	− 4.59	<.0001
*group*		0.10	0.05	2.02	0.044

**Fig. 6:**
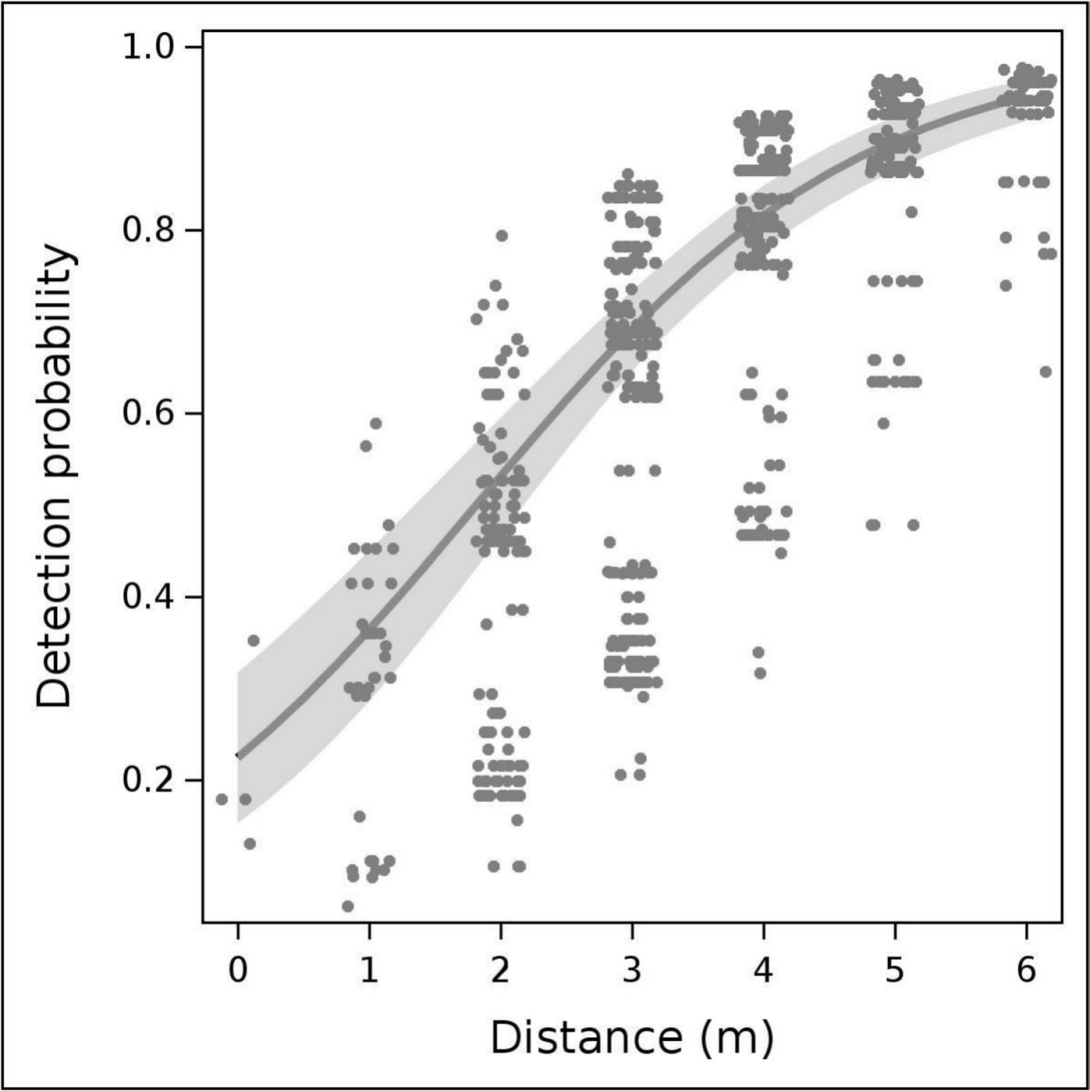
The graph shows the detection probability of wild boar (ordinate) as a function of distance (abscissa) in the Castelporziano Preserve (Rome, Italy). The dots represent the predicted values of individual observations, the continuous line represents the predicted mean value, and the grey band refers to the 95% confidence interval

The prediction plot (Fig. 6) illustrates the relationship between detection probability (*p*_*EXP*_) and *distance*, revealing that the detection probability is lowest near the test CT but never reaches 1, though it is very high beyond 4 m. The average detection probability (*p*_*EXP*_) was 0.60 ± 0.25. The selected model is robust, as shown in Supplementary Figures S1 and S2.

**Fig. 7:**
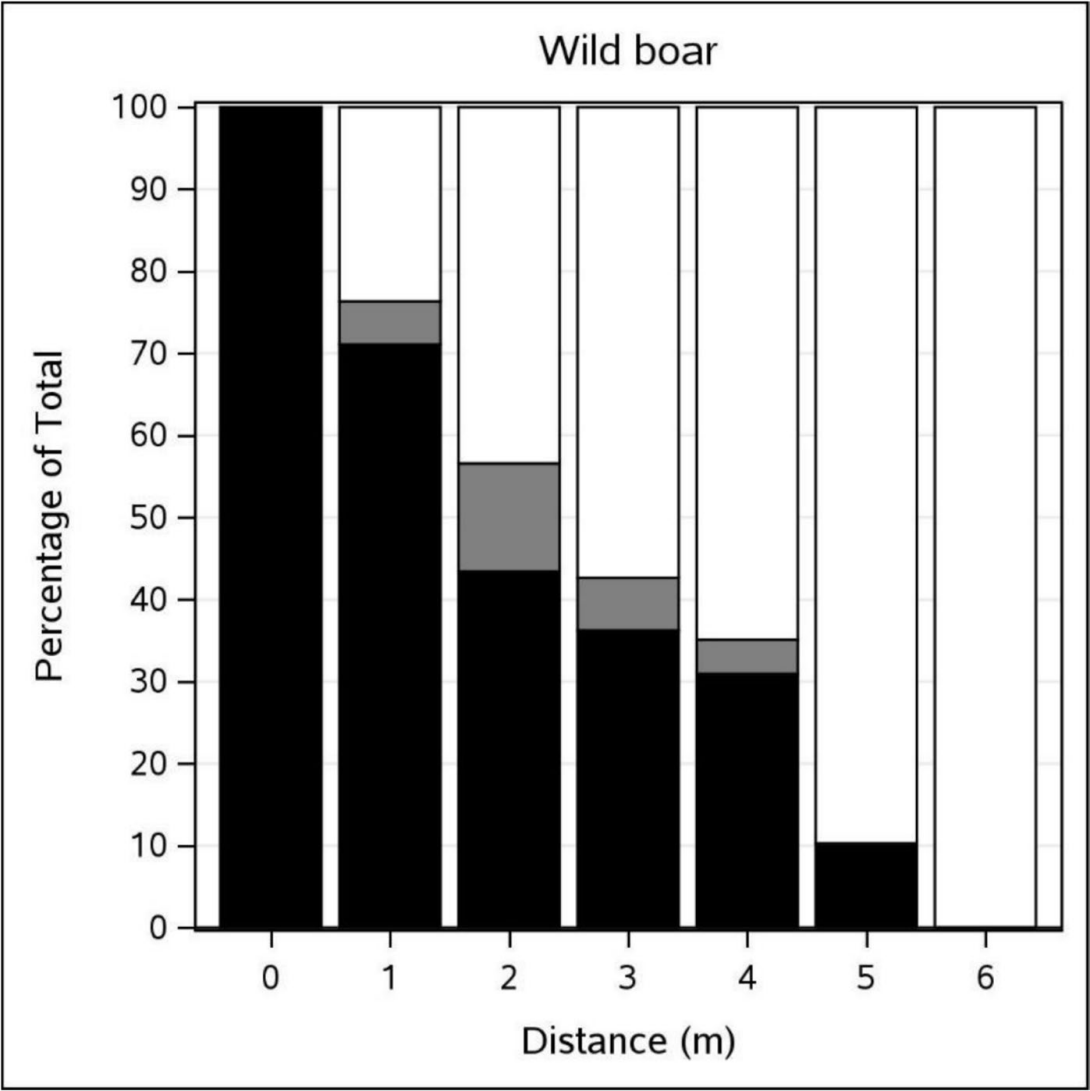
Stacked 100% bar chart showing the fraction of wild boar groups missed (black), partially detected (grey) or fully detected (white) by the test CT at increased distances. The ordinate shows the fraction of wild boar groups, and the abscissa shows the distance from the CT.

The histogram (Fig. 7) confirms that up to 4 m, many animals are missed or groups are only partially detected, but detection improves markedly at 5–6 m.

**Fig. 8:**
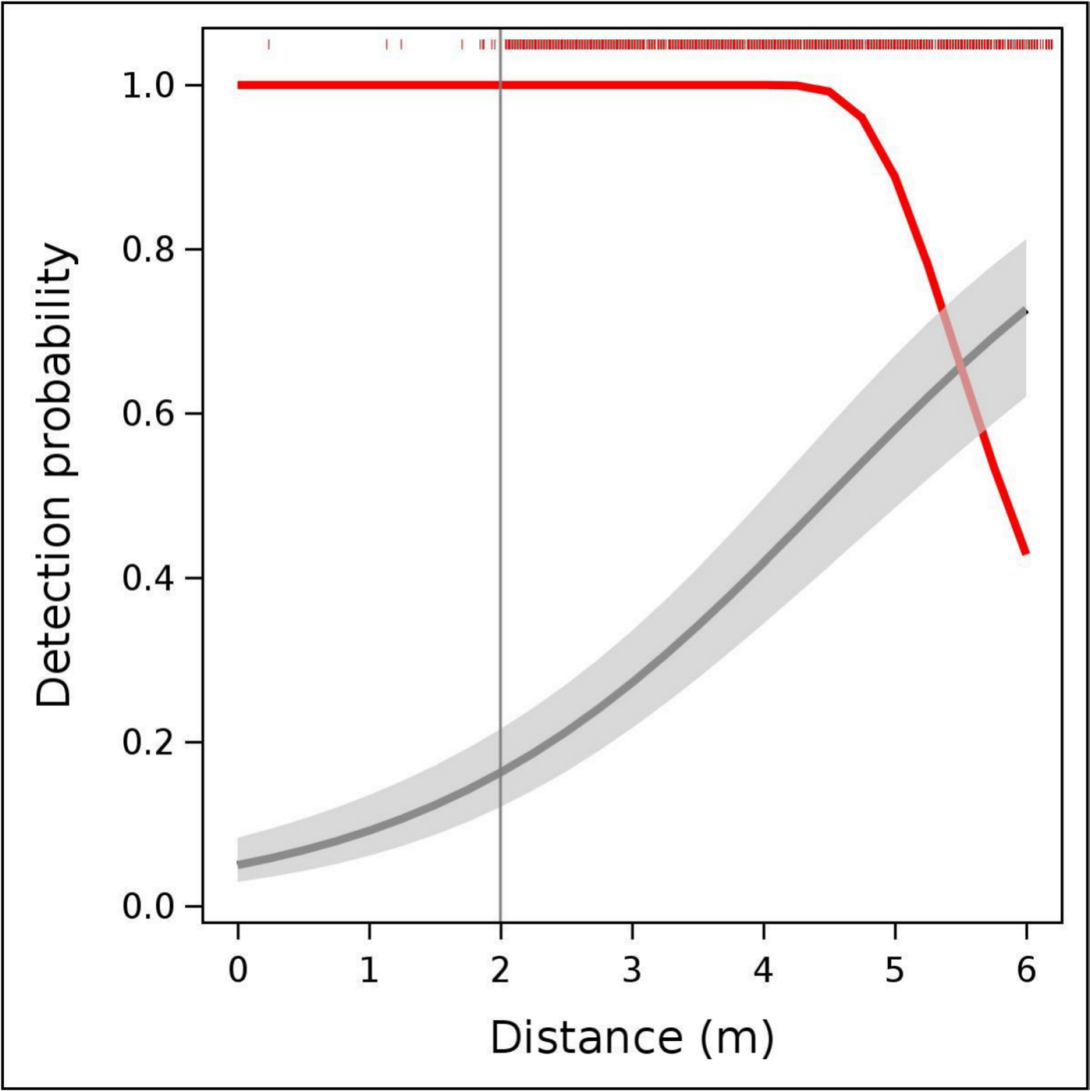
We present the detection function obtained from the experiments (black continuous line) alongside its 95% confidence limit (grey band), and compare it with the detection function estimated for the survey (red line). The upper red vertical bars represent the distance from the CT of the individual observations collected during the survey

The detection functions derived from the survey were the hazard rate function which is compared with the one derived from the experiment in Fig. 8. The former assumes certain detectability from 0 to 4.5 m whereas the experimental function shows that wild boars are nearly undetectable at distances between 0 and 2 m.

The survey yielded a daily range of 9.89 ± 0.42 km, an effective detection angle of 42.48 ± 0.23°, and a trapping rate of 2.24 ± 0.65 animals/day, resulting in a detection probability *p*_*SUR*_ = 0.88 ± 0.009 and an effective detection radius *r*_*SUR*_ = 5.63 ± 0.06 m. The survey data yielded a density of 46.13 ± 13.61 wild boar/km^2^. In contrast, our experiment produced *p*_*EXP*_ = 0.60 ± 0.01 and *r*_*EXP*_ = 4.65 ± 0.5 m. Using these values, the experiment estimated 55.64 ± 16.20 wild boar/km^2^. The two estimates were significantly different (Student’s test, *t* = 12.42, *P* < 0.001). We concluded that the survey underestimated the density of 17% because of a lack of certain detectability near the CT.

The comparison of the three CT models (Uovision, Bolyguard and Browning Spec Ops HP5) showed no significant differences across the three dependent variables: group size (*χ*^2^_2_ = 3.28, *P* = 0.19), sequence duration (*χ*^2^_2_ = 0.19, *P* = 0.91), and number of detections (*χ*^2^_2_ = 3.77, *P* = 0.15).

## Discussion

This study presents a validation framework for a critical assumption underlying many CT population estimation methods: perfect detectability near the CT.

Using the wild boar as a case study, we observed that CTs failed to detect a significant fraction of animals moving close to the device, leading to a 17% underestimation of population size when using REM, higher than estimates reported by McIntyre et al., (2020) which ranged around 6–10%. Consequently, we highly recommend to undertake a pilot study to check the performance of CTs and whether the assumptions of the selected assessment method can be met in the field. While standardized protocols for data collection are useful, the complexity of natural environments and the variable influence of anthropogenic stressors can still affect detection rates.

We also found that CT technology itself plays a minor role in the detection process. Using a large number of high-grade CTs may not be feasible for wildlife managers facing financial constraints. The protocol outlined in experiment 3 offers a practical method for selecting a cost-effective CT model tailored to specific study conditions.

Previous studies (Kays et al., 2021; Nakashima et al., 2022; Rowcliffe et al., 2011; Wong et al., 2019) have acknowledged imperfect CT detection, prompting corrections based on distance sampling theory. However, these approaches assume perfect detection near the CT, an assumption that our study challenges. We found that the detection probability of wild boar increases with distance from 0 to ~4 m outward, contradicting standard models and posing significant problems when using CT-based methods. Although Rowcliffe et al., (2011) and DeWitt & Cocksedge (2023) reported similar effects, this issue has often been overlooked in the literature, although it has important implications for accurate population estimates. For such cases, Mark-Recapture Distance Sampling (MRDS, Manly et al., 1996) offers a potential solution to estimate *h*(0).

The difference in detectability between day and night was significant and consistent with the findings of Palencia et al., (2021). Differences in detectability across habitats may be linked to vegetation thickness (Kolowski et al., 2021) or varying animal movement patterns in different habitats, because they provide more food or better cover. This could explain our unexpected finding that detection probability was inversely related to the local canopy obstruction (*CanOb)*. This outcome aligns with the negative coefficient observed for old-growth pine forests (habitat 4). Despite their high *CanOb* values, these habitats are of low quality and are likely perceived as unsafe by the species due to the lack of undergrowth and shelters. This sparse vegetation and the scarcity of food may have caused animals to move through quickly, reducing their chances of being detected by a CT.

Enhancing CT quality or implementing the double-observer method (Nakashima et al., 2022; Nichols et al., 2000) could improve detectability, but both options come with increased costs. High-grade CTs demand substantial financial investments, while the double-observer method requires doubling the number of CTs deployed and additional labour is required. These trade-offs often make such solutions impractical for routine wildlife monitoring. Therefore, we reiterate the need for managers or practitioners conducting pilot studies, ensuring the most cost-effective approach is identified for their specific research context.

Our study design has a few limitations that should be addressed in future research. The first potential issue is the presence of blind areas, as shown in Fig. 2. These areas, which the camera traps did not fully cover, may have led to a potential overestimation of overall detectability. The second potential bias relates to our experimental setup. The presence of four CTs and stakes could have influenced animal behavior, potentially lowering detection rates for a shy species or increasing them for curious ones. While we were unable to fully investigate the effects of these biases due to our limited number of available CTs, future studies should explore them in greater detail. For the wild boar, the risk of missing animals using three control CTs was just 2.5%.

In conclusion, while CT methods remain powerful tools for wildlife monitoring, our findings demonstrate that perfect detectability near CTs cannot be assumed. Without rigorous pilot studies and standardized validation protocols, widely used approaches like REM risk significantly underestimating population densities. Consequently, managers must adapt CT deployment and analysis to their specific ecological context, carefully balancing practical field constraints with scientific rigor. Where such rigor cannot be fully maintained, any resulting estimates must be interpreted with due caution.

## Supplementary Information

Below is the link to the electronic supplementary material.

ESM 1 DOCX (141 KB)

## Data Availability

All data supporting the findings of this study will be made available on Dryad upon publication.
