# Supplementary material for: Short-distance detectability in camera trap surveys: implications for population assessment
Source: Environ Monit Assess. 2026 Apr 17;198(5):473. doi: 10.1007/s10661-026-15272-7 (PMC13090299; doi:10.1007/s10661-026-15272-7)
Supplement: Supplementary file 1 — DOCX (141 KB) [file 10661_2026_15272_MOESM1_ESM.docx]

**SUPPLEMENTARY MATERIALS**

The plot of Fig. S1 allows to evaluate the quality of the selected model 1 for the wild boar. The distribution of residuals is almost normal and it is centred around zero. The substantially good fitting is demonstrated by the QQ plot in Fig. S2 where residuals are well aligned along the normal line.


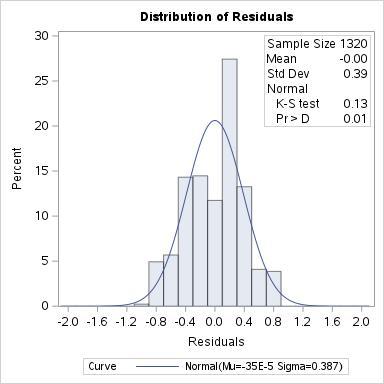


**Figure S1**. distribution of residuals of model 1 relative to the wild boar.


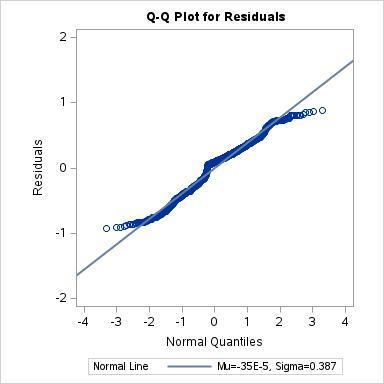


**Figure S2**. QQ plot of residuals of model 1 relative to the wild boar. points are well overlapped with the 1:1 line from -2 to 2. i.e. for the largest part of available x range. We only note some bias in the tails, probably justified by the small number of values in this region and maybe there is the presence of a spike at x=-0.2. .
